# Microbiota-derived metabolites and cognitive dysfunction in dialysis patients: mechanisms and targeted therapeutic strategies

**DOI:** 10.3389/fmicb.2026.1878545

**Published:** 2026-07-13

**Authors:** Jun Xiao, Tian Xu, Yanfang Fu, Jing Xia, Zhiying Chen, Xiaoping Yin, Xulong Chen

**Affiliations:** 1 Department of Neurology, Jiujiang University Affiliated Hospital, Jiujiang, Jiangxi, China; 2 The First Clinical College of Gannan Medical University, Ganzhou, Jiangxi, China; 3 School of Clinical Medical, Jiujiang University, Jiujiang, Jiangxi, China

**Keywords:** chronic kidney disease, cognitive dysfunction, dialysis, gut microbiota, microbiota-gut-brain-kidney axis

## Abstract

Cognitive impairment (CI) in dialysis patients is a common and serious complication that significantly affects prognosis, with lack of effective treatment strategies. However, its mechanisms are still not fully clear, and effective treatment strategies are still limited. In recent years, more evidence has suggested that gut microbiota dysbiosis and changes in gut-derived metabolites may be involved in the development of CI in dialysis patients through the microbiota-gut-kidney-brain axis. Recent studies have shown that gut microbiota dysbiosis in dialysis patients may promote the progression of CI through several pathways. These include the accumulation of gut-derived uremic toxins, such as indoxyl sulfate (IS), p-cresyl sulfate (PCS), and Trimethylamine N oxide (TMAO), changes in bile acid metabolism; and the reduction of short-chain fatty acids (SCFAs) with neuroprotective effects. These changes may damage the intestinal barrier and the blood–brain barrier (BBB), and promote systemic inflammation, oxidative stress, and neuroinflammation. As a result, cognitive dysfunction in dialysis patients may be further aggravated. Therefore, targeting the gut microbiota has become a promising treatment direction. These strategies include dietary intervention, probiotics and related preparations, fecal microbiota transplantation (FMT), and targeted removal of uremic toxins and their derivatives. This review summarizes the gut microbiota composition associated with CI in dialysis patients, examines the molecular mechanisms of injury mediated by the microbiota–gut–brain–kidney axis, evaluates current microbiota-targeted interventions, and discusses future research directions for improving clinical prevention and treatment.

## 1 Introduction

Approximately 3.6 million people worldwide are receiving dialysis. About 89% of them receive hemodialysis (HD), and about 11% receive peritoneal dialysis (PD) (GBD 2023 Kidney Failure with Replacement Therapy Collaborators, 2025). Although dialysis is an important life-sustaining treatment for patients with kidney failure, long-term dialysis is still associated with many complications. These include cardiovascular disease, malnutrition, chronic inflammation, and neurological damage. Among these complications, cognitive impairment (CI) is a common neurological complication in patients with chronic kidney disease (CKD), especially in those receiving maintenance dialysis (McIntyre and Jain, 2025). CI is mainly characterized by impairment in one or more key brain functions, such as memory, learning, attention, and decision-making. Severe CI is usually referred to as dementia (American Psychiatric Association, 2022). The burden of CI is particularly high in dialysis patients. Its prevalence is about 40–50%, which is higher than that in non-dialysis patients 32% and kidney transplant recipients 26% (Zhang et al., 2024). Cognitive impairment not only reduces quality of life and treatment adherence in dialysis patients, but also increases family burden and healthcare burden. It may also raise ethical issues, such as eligibility for kidney transplantation (Farisco et al., 2024).

The occurrence of CI in dialysis patients cannot be fully explained by traditional risk factors for vascular or neurodegenerative diseases. Older age, diabetes, hypertension, depression, sleep disorders, and cerebral small vessel disease are common in dialysis patients. These factors may all be involved in cognitive decline (Crowe et al., 2021; Lau et al., 2017). However, the high burden of dialysis-associated cognitive impairment (DACI) patients suggests that End-Stage Renal Disease (ESRD)-related metabolic disorders and dialysis-related physiological stress may also play important roles. Uremic toxin retention, chronic low-grade inflammation, oxidative stress, hemodynamic instability during dialysis, endothelial dysfunction, and blood–brain barrier injury may interact with each other. Together, these changes may promote cerebrovascular injury and neuroinflammation, and may finally contribute to cognitive decline (Capasso et al., 2025; Andrews et al., 2025).

In recent years, the microbiota–gut–brain–kidney axis (Zhu et al., 2025) has become a potential framework for understanding the interaction among gut dysbiosis, uremic toxicity, systemic inflammation, and neurological injury. In the context of CKD or ESRD, gut dysbiosis may change microbial metabolism. It may increase the production of gut-derived uremic toxins (UTs), especially protein-bound uremic toxin (PBUT). It may also reduce protective metabolites such as short-chain fatty acids (SCFAs), increase Lipopolysaccharide (LPS)-related inflammatory burden, and impair intestinal barrier integrity (Mishima et al., 2017). When CKD progresses to dialysis treatment, uremic toxin retention, dietary restriction, medication use, and persistent low-grade inflammation may further worsen gut dysbiosis (Rysz et al., 2021). These pathological changes may jointly worsen kidney function and cognitive function. They may form a harmful interaction among the gut, brain, and kidney (Zhu et al., 2025). This provides biological plausibility for the link between gut microecological disturbance and CI in dialysis patients. At present, effective treatments for CI in dialysis patients are still lacking. Therefore, emerging therapies targeting gut microbiota and their metabolites may offer new opportunities for prevention or treatment of cognitive decline in this population.

This review summarizes the characteristic changes in gut microbiota and their metabolites in dialysis patients. It also reviews the evidence linking these changes to cognitive phenotypes. In addition, this review discusses potential preventive and therapeutic strategies targeting gut microbiota and their metabolites. However, high-quality clinical evidence directly examining the relationship between gut dysbiosis and CI in dialysis patients is still limited. Most current studies are cross-sectional studies, animal experiments, or *in vitro* studies. Therefore, this review integrates evidence from different fields. The discussion of mechanisms is mainly based on inference from existing studies. It should not be regarded as a definite causal explanation. Thus, this review does not aim to propose a confirmed causal model. Instead, it aims to outline biologically plausible pathways, identify current controversies and evidence gaps, and provide future research directions for the prevention and management of dialysis-associated cognitive impairment.

## 2 Alterations in the gut microbiota and its metabolites in patients undergoing dialysis and their potential neurocognitive relevance

### 2.1 Alterations in the gut microbiota in patients undergoing dialysis

Gut microbiota dysbiosis is common in patients with CKD and in patients receiving dialysis (Lin et al., 2021; Bao et al., 2022; Wang H. et al., 2023). After patients progress to the dialysis stage, gut microbiota dysbiosis may become more pronounced in both HD and PD patients. Several studies have reported reduced gut microbiota diversity in dialysis patients, which may be associated with poor clinical outcomes (Li Y. et al., 2019; Wu et al., 2023). A clinical study of patients with ESRD undergoing HD showed that lower gut microbial *α* diversity was associated with higher mortality. In that study, the risk of death was significantly lower in the high-*α*-diversity group than in the low-α-diversity group (Lin et al., 2021). Similarly, a clinical study of PD patients found that reduced gut microbial α diversity was associated with poor prognosis and was independently related to a higher risk of PD technique failure (Guo et al., 2023). Studies have shown that when CKD progresses to ESRD and patients enter the dialysis stage, the gut microbial community shows more obvious changes in structure and composition (Zhao et al., 2021b; Shi et al., 2022). One clinical study showed that bacteria related to UTs production, for example *Bacteroides*, were predominant in CKD patients gut microbiome. The gut microbial profiles differed between dialysis modalities, with *Bacteroidetes* and *Fusobacteria* being relatively abundant in PD patients, while HD patients exhibited a higher predominance of *Proteobacteria* and *Verrucomicrobia* (Shivani et al., 2022). A recent metagenomic study in non-dialysis CKD patients also showed clear alterations in the gut microbiome when contrasted with healthy controls. These changes were described as a “toxic microbiome” pattern and became more obvious as kidney function worsened. In patients with advanced CKD, bacteria carrying genes related to UTs precursor production, such as *Desulfovibrio fairfieldensis*, *Bacteroides clarus*, and *Blautia obeum*, were more enriched. In contrast, bacteria related to SCFAs production and gut mucosal homeostasis, such as *Anaerobutyricum hallii*, *Romboutsia timonensis*, and *Intestinibacter bartlettii*, were significantly reduced. These changes were positively associated with increased levels of indole- and phenol-type PBUT, such as indoxyl sulfate (IS), p-cresyl sulfate (PCS) (Laiola et al., 2025). It is also worth noting that a similar pattern of “decreased protective commensal bacteria and increased toxin-producing or pro-inflammatory bacteria” has been observed in PD patients (Wu I.-W. et al., 2020; Tian N. et al., 2023). Similarly, reduced gut microbial *α* diversity has been observed in patients receiving continuous ambulatory peritoneal dialysis (CAPD), compared with healthy controls and patients with ESRD who were not receiving dialysis. In addition, the microbial community showed a distinct structural shift. Compared with healthy controls, patients receiving CAPD showed a reduced abundance of beneficial commensal bacteria involved in SCFAs SCFAs, especially butyrate, such as *Bacteroidaceae*/*Bacteroides* and *Enterococcus faecalis*, were decreased. In contrast, opportunistic pathogens that produce LPS and UTs, such as *Proteobacteria*, *Enterobacteriaceae*, and *Escherichia-Shigella*, were increased (Li et al., 2024). These findings suggest that the gut ecological niche may shift toward a more pro-inflammatory and pro-infectious state. Similar features have also been observed in patients with HD-ESRD. Compared with the healthy control group, gut bacteria associated with UTs production, such as *Eggerthella lenta*, *Flavonifractor*, *Alistipes*, *Ruminococcus*, and *Fusobacterium*, were relatively enriched. In contrast, SCFAs-producing bacteria, including *Prevotella copri* (*P. copri*), *Clostridium* spp., *Roseburia*, *Faecalibacterium prausnitzii*, and *Eubacterium rectale*, were reduced. These changes were accompanied by increased levels of UTs such as IS and PCS (Wang et al., 2020). Furthermore, a larger deep metagenomic study further demonstrated broad functional shifts in the gut microbiome of HD-ESRD patients when compared with healthy controls. The gut metabolic profile shifted from a pattern mainly related to SCFAs metabolism to a pattern with increased metabolism related to UTs and secondary bile acids (SBAs). Classic SCFAs-producing bacteria, represented by *Prevotella* and *Roseburia*, showed a significant decline. In contrast, bacteria related to UTs production, such as *Blautia*, *Dorea*, and *Eggerthellaceae*, and species related to SBAs production, such as *Dorea* and *Hungatella* from the *Lachnospiraceae*, as well as *Actinobacteria* representatives including *Collinsella intestinalis* and *E. lenta*, were significantly enriched (Zhang et al., 2023).

As CKD progresses to ESRD and the dialysis stage, gut dysbiosis is not limited to changes in bacterial composition. The gut mycobiome also changes significantly. This change is characterized by a decrease in commensal or food-derived fungi, such as *Saccharomyces cerevisiae*, and an increase in opportunistic pathogens, such as *Candida*, *Aspergillus fumigatus*, *Cladophialophora immunda*, *Exophiala spinifera*, *Hortaea werneckii*, and *Trichophyton rubrum* (Ren et al., 2024). In contrast, in the earlier stages of CKD, the gut fungal profile shows an increase in *Apiotrichum* and *Saccharomyces*, while opportunistic or pathogenic fungi, such as *Candida*, *Rhodotorula*, *Ganoderma*, and the white-rot fungus-related genus *Bjerkandera*, are decreased (Qiu J. et al., 2023). Taken together, with the continuous decline in kidney function and the influence of treatment-related factors, the gut mycobiome may gradually shift from a relatively commensal- or food-derived fungus-dominant state to an opportunistic pathogen-dominant state. Notably, the enrichment of opportunistic pathogens is positively associated with the level of serum creatinine, homocysteine (Hcy), and phenylacetylglycine. In contrast, *Saccharomyces* is negatively associated with fecal toxic metabolite levels and kidney function decline. Collectively, these results suggest a potential role of gut fungal imbalance in the development of metabolic abnormalities associated with ESRD (Ren et al., 2024; Qiu J. et al., 2023; Hu J. et al., 2022).

In addition, accumulating evidence indicates that gut microbiota may contribute to the development and progression of DACI through several gut–kidney–brain-related pathways (Liang et al., 2022; Gao et al., 2024; Zhu et al., 2020; Wang J. et al., 2023). In general, individuals with cognitive impairment often show a decrease in bacteria related to the production of SCFAs, such as *Odoribacter*, *Butyricimonas*, and *Bacteroides* (Liang et al., 2022). Among them, the abundance of *Odoribacter* is positively associated with brain structural indicators, such as white matter volume and right hippocampal volume. This suggests a potential link between insufficient protective metabolic output and increased brain vulnerability (Liang et al., 2022). In patients receiving maintenance hemodialysis (MHD), clinical studies provide evidence for a potential relationship between gut microbial alterations and cognitive function. SCFAs-producing genera, such as *Roseburia*, *Faecalibacterium*, and *Bifidobacterium*, are significantly reduced and exhibit a positive correlation with the total score of the Montreal Cognitive Assessment (MoCA) or with specific cognitive domains (Gao et al., 2024). Another study found that patients with mild cognitive decline undergoing HD had significant changes in microbial diversity and several bacterial genera. This study also suggested that bile acid-metabolizing bacteria, such as *Bilophila*, and serum putrescine may be candidate markers for HD-related mild cognitive decline (Zhu et al., 2020). Similar depletion of “protective functional bacteria” has also been observed in patients undergoing PD. Notably, bacteria associated with lactate production, SCFA synthesis, and resistant starch degradation, represented by *Bifidobacterium*, *Butyricicoccus*, and *Ruminococcus 2*, respectively, were significantly reduced. These findings suggest that dialysis-related factors may further weaken carbohydrate fermentation and the maintenance of mucosal homeostasis. This may promote inflammation, increase barrier vulnerability, and contribute to neurocognitive decline (Wang J. et al., 2023).

Overall, although there are differences in the composition of the gut microbiota between patients with ESRD undergoing HD and those undergoing PD, both groups show signs of gut dysbiosis. This remodeling is characterized by decreased alpha diversity and community stability, reduced dominant SCFAs-producing commensal bacteria, expansion of opportunistic pathogens or potential toxin-producing bacteria, increased oxidative stress, and enhanced metabolic pathways related to UTs, LPS, protein and nucleotide metabolism, and bile acid metabolism. These findings provide microbiome-level evidence for explaining dialysis-associated cognitive decline from the perspective of the gut–brain–kidney axis.

### 2.2 Alterations in gut-derived metabolite profiles in dialysis patients and their potential associations with cognitive impairment

Growing metagenomic functional evidence suggests that, with the progression of CKD, gut microbial metabolism shifts from mainly carbohydrate fermentation to protein and nucleotide metabolism (Laiola et al., 2025; Li et al., 2024; Krukowski et al., 2026). This shift leads to disorders of several major gut-derived metabolites in plasma, including increased UTs, disturbed bile acid metabolism, and decreased SCFAs. Specific gut-derived metabolites are shown in Table 1. These changes are also accompanied by increased oxidative stress, impaired gut barrier function, and increased endotoxin burden. In common, these gut-derived metabolites can be eliminated through glomerular filtration and tubular secretion. As CKD progresses, renal excretory capacity gradually declines. This decline reduces the clearance of gut-derived UTs. As a result, these toxins accumulate in the circulation and contribute to a pro-inflammatory and pro-oxidative internal environment. Patients requiring dialysis usually have more advanced kidney failure. They often have a higher burden of UTs and systemic inflammation than patients with non-dialysis CKD. However, conventional dialysis cannot adequately remove PBUT (Gryp et al., 2020). In general, PBUT are difficult to cross the blood–brain barrier (BBB). However, CKD patients who require dialysis often have impaired albumin-binding capacity. This may increase the proportion of free UTs, damage the BBB, and allow these toxins to enter the brain. After entering the brain, they may injure neural progenitor cells, blood vessels, and monoaminergic neurons (Capasso et al., 2025). This mechanism is supported by imaging studies. IS and indole-3-acetic acid (IAA) may trigger the aryl hydrocarbon receptor (AhR), which may mediate BBB disruption and cognitive dysfunction (Wu P.-H. et al., 2020; Wu P.-H. et al., 2024). At the same time, different dialysis modalities may also affect the composition of the gut microbiota and may be further related to changes in cognitive function. Compared with PD, HD is more likely to cause hemodynamic instability and blood pressure fluctuations during treatment, which may increase the risk of cerebral hypoperfusion and cognitive impairment (Drew et al., 2019). In addition, studies have found that serum PCS levels are lower in PD patients than in HD patients. This suggests that differences in gut microbiota and metabolic features related to different dialysis modalities may contribute to the UTs burden and its potential effects on cognitive function (Lu M. et al., 2026). Therefore, it is important to clarify how dialysis affects gut-derived metabolism and how these changes influence cognitive function in patients with CKD.

**Table 1:** Alterations in gut microbiota and microbial metabolites in dialysis patients.

Study	Cohort and control	Sequencing	Microbiota	Metabolites	ALTERATION
Li Y. et al. (2019)	CKD5-HD *n* = 29 vs. healthy controls *n* = 69	16S rRNA	*Neisseria, Lachnoclostridium,* and *Bifidobacterium*	IS, PCS	↑
			*Faecalibacterium*	/	↓
Wang et al. (2020)	HD *n* = 233 vs. healthy controls *n* = 69	Shotgun metagenomics	*Eggerthella lenta, Flavonifractor, Alistipes, Ruminococcus,* and *Fusobacterium*	IS, PCS, PAG, phenol, hippuric acid, TMAO, SBAs	↑
			*Prevotella* spp.*, Clostridium* spp.*, Roseburia* spp.*, Faecalibacterium prausnitzii,* and *Eubacterium rectale*	SCFAs, PBAs	↓
Zhang et al. (2023)	HD *n* = 378 vs. healthy controls *n* = 290	Shotgun metagenomics	*Blautia* spp.*, Dorea* spp., and *the family Eggerthellaceae*	IS, PCS, PAG, TMAO, SBAs	↑
			*Prevotella* and *Roseburia*	SCFAs	↓
Li et al. (2024)	CAPD *n* = 72 vs. healthy controls *n* = 13	16S rRNA + metabolomics		PCS, TMAO, D-lactate	↑
			*Bacteroidaceae, Bacteroides,* and *Faecalibacterium*	BAs, CA	↓
Wu I.-W. et al. (2020)	PD *n* = 76 Vs. CKD *n* = 22	16S rRNA	*Lactobacillus, Escherichia_Shigella,* and *Collinsella stercoris*	IS, PCS	↑
			*Dialister, Lachnospiraceae_ND3007_group, Pseudobutyrivibrio, Roseburia, Paraprevotella, Ruminiclostridium,* and *Bacteroides eggerthii*	/	↓
Lin et al. (2021)	Dead *n* = 15 Vs. Alive *n* = 94	16S rRNA	*Bacteroidetes*	/	↑
			*Succinivibrio* and *Anaerostipes*	/	↓
Shi et al. (2022)	HD *n* = 24 Vs. healthy controls *n* = 16	Shotgun metagenomics	*Bacteroides fragilis, Clostridium,* and *Ruminococcus torques*	*/*	↑
			*Dialister, Barnesiella,* and *Coprococcus*	/	↓
Wu et al. (2023)	HD *n* = 96 Vs. healthy controls *n* = 81	16S rRNA	*Escherichia, Streptococcus, Lactobacillus, Enterococcus, Staphylococcus,* and *Klebsiella*	/	↑
			*Bifidobacterium, Prevotella,* and *Bacteroides*	/	↓
Lv et al. (2025)	MHD *n* = 45 Vs. healthy controls *n* = 30	16S rRNA	*Escherichia-Shigella, Blautia,* and *Citrobacter*	/	↑
			*Prevotella copri, Bacteroides vulgatus,* and *Agathobacter*	SCFAs	↓
Kuo et al. (2025)	HD n = 85	16S rRNA	*Bacteroides* and *Prevotella*	/	↑
Guo et al. (2023)	PD *n* = 101	16S rRNA	In the low-diversity group: *Escherichia-Shigella, Enterobacteriaceae, and unclassified Bacteria* In the high-diversity group: *Bacteroides, Lachnospiraceae,* and *Blautia*	/	↑
Bao et al. (2022)	CAPD *n* = 105 Vs. healthy controls *n* = 102	16S rRNA	*Escherichia-Shigella, Flavonifractor, Fusobacterium, Clostridium, Eubacterium,* and *Bacteroides*	IS, PCS, TMAO	↑
			*Bifidobacterium, Faecalibacterium, Subdoligranulum,* and *Roseburia*	/	↓
Tian N. et al. (2023)	CAPD *n* = 30	16S rRNA	*Akkermansia, Peptostreptococcaceae incertae sedis, Megasphaera,* and *Coprobacter*	/	↑
			*Defluviitaleaceae_uncultured,* and *Ruminococcaceae_incertae_sedis*	SCFAs	↓
Wang J. et al. (2023)	PD *n* = 28 Vs. non-dialysis ESRD *n* = 29	16S rRNA	*Prevotellaceae, Fusobacteria, and Proteobacteria*	/	↑
			*Lactobacillaceae, Actinomycetaceae, Actinomyces, Streptococcaceae, Streptococcus, Atopobium,* and *Propionibacteriaceae*	/	↓
Lu M. et al. (2026)	CAPD *n* = 50 Vs. HD *n* = 50	16S rRNA	*Fusobacteriota, Fusobacterium,* and *Escherichia-shigella*	*/*	↑
			*Bacteroides, Prevotella, Subdoligranulum,* and *Faecalibacterium*	PCS, IS	↓

↑ indicates increased abundance or concentration; ↓ indicates decreased abundance or concentration. Taxa are presented at the genus or species level as reported in the original studies. Bacterial names are italicized. Spp., species pluralis, indicating multiple species within a genus. CKD5-HD, stage 5 chronic kidney disease patients undergoing hemodialysis; HD, hemodialysis; PD, peritoneal dialysis; CAPD, continuous ambulatory peritoneal dialysis; MHD, maintenance hemodialysis; ESRD, end-stage renal disease; 16S rRNA, 16S ribosomal RNA; IS, indoxyl sulfate; PCS, p-cresyl sulfate; PAG, phenylacetylglutamine; TMAO, trimethylamine N-oxide; SBAs, secondary bile acids; SCFAs, short-chain fatty acids; PBAs, primary bile acids; BAs, bile acids; CA, cholic acid.

PBUT is one of the most representative metabolic abnormalities in dialysis patients. PBUT is mainly represented by IS, PCS, IAA, and Hcy. A key feature of these compounds is their strong protein-binding capacity, which limits their removal during dialysis. In addition, their UTs precursors are mainly produced through the metabolism of aromatic amino acids by the gut microbiota (Zhang et al., 2023). High levels of PBUT in patients with ESRD who require dialysis may have negative effects on gut microbiota, kidney function, and neurocognitive function through oxidative stress, neuroinflammation, BBB disruption, and the gut–brain–kidney axis (Zhu et al., 2025; Bao et al., 2022; Laiola et al., 2025; Glorieux et al., 2023; Faucher et al., 2023). Specifically, increased blood levels of IS and PCS in patients with ESRD who require dialysis are linked to higher levels of biomarkers related to inflammation and oxidative stress, such as interleukin-6 (IL-6) and glutathione peroxidase (GPx), as well as gut microbiota dysbiosis (Li Y. et al., 2019; Rossi et al., 2014). Enhanced amino acid metabolic activity may also be involved in this process (Lin et al., 2022). Similarly, animal studies have shown that IS and IAA can induce oxidative stress injury in hippocampal neurons, leading to impaired learning and memory. Collectively, current evidence supports the possibility that PBUT contribute to the pathogenesis of CKD-related cognitive impairment (Watanabe et al., 2021).

IS can increase BBB permeability by disrupting tight junctions in the BBB and then enter the brain. It can reduce neuronal activity and glutathione levels, and then induce inflammation, oxidative stress, and cell death. These effects may contribute to CKD-related cognitive impairment (Capasso et al., 2025; Jeong et al., 2024). In patients with CKD, IS is associated with cognitive decline, while PCS shows no significant association with cognitive decline (Yeh et al., 2016). In HD patients, IS is also associated with cognitive impairment, and its blood concentration is negatively correlated with cognitive function (Lin et al., 2019b). In addition, one clinical study in HD patients showed that reducing blood levels of IS and Hcy could improve cognitive function (Wang et al., 2025).

PCS often remains at a high level during the dialysis stage. However, current clinical evidence indicates that the relationship between PCS and cognitive impairment appears to be less pronounced than that observed for IS (Lin et al., 2019b). Nevertheless, PCS may still indirectly contribute to DACI through the vascular–inflammatory pathway. In HD patients who had received dialysis for more than 6 months, high plasma PCS levels were linked to a higher risk of ischemic stroke (Tan et al., 2022). Interestingly, one study reported lower serum PCS levels in patients undergoing PD than in those receiving HD, which might be linked to differences in microbial metabolite profiles between dialysis modalities (Lu M. et al., 2026). In nephrectomized mice, reducing serum PCS levels decreased oxidative stress and neuroinflammation, and improved neurological changes such as depression, anxiety, and cognitive impairment (Sun et al., 2020). In addition, PCS may weaken BBB integrity by affecting tight junction proteins and transporter function (Faucher et al., 2023), thereby having adverse effects on cognitive function.

In dialysis patients, IAA is a PBUT produced from tryptophan metabolism by the gut microbiota. Increased IAA levels are considered to be related to neurocognitive outcomes. The BREIN indicated that IAA levels were significantly increased in patients with ESRD compared with healthy individuals, suggesting that increased exposure to indole-derived UTs may coexist with cognitive impairment (Bobot et al., 2024). A cohort study in HD patients showed that serum IAA was associated with cognitive impairment. It could increase the risk of cognitive impairment and was related to lower cognitive scale scores (Lin et al., 2019a). Studies in HD patients also found that blood IAA levels were associated with the enrichment of UTs-producing bacteria, such as *Bacteroides thetaiotaomicron* and *Fusobacterium* var*ium*. These studies further suggested that IAA may activate the AhR, contribute to vascular endothelial injury, systemic inflammation, and oxidative stress, which may further elevate cardiovascular and cognitive risks (Wu P.-H. et al., 2020; Wu P.-H. et al., 2024).

Hcy may also indirectly contribute to DACI through the vascular–inflammatory pathway. In a study of patients with type 2 diabetes, increased Hcy mediated the association between kidney function decline and cognitive impairment through endothelial dysfunction and increased cerebral small vessel disease burden (Sonoda et al., 2017). A study in HD patients found that IS and Hcy were independent risk factors for cognitive impairment. It also showed that, with stronger combined dialysis strategies, IS and Hcy levels decreased more clearly, while MMSE and MoCA scores improved in a stepwise manner (Wang et al., 2025). However, it should be emphasized that high Hcy levels are not always directly related to cognitive decline. Related randomized controlled trials showed that although B vitamin supplementation reduced plasma total Hcy levels, it did not significantly improve cognitive outcomes (Brady et al., 2009; Scott et al., 2017).

Overall, increased levels of IS, PCS, IAA, and Hcy point to a uremic environment characterized by increased exposure to gut-derived metabolites and limited removal by dialysis. This environment may disrupt the BBB and affect cognitive function. Among these metabolites, the evidence linking IS to cognitive decline is relatively more consistent. PCS and Hcy may mainly act through the vascular–inflammatory pathway, while IAA suggests that indole-derived metabolites may need to be evaluated as a whole.

Second, gut-derived uremic toxins are not limited to PBUT; other metabolites, such as trimethylamine N-oxide (TMAO), PAG, and hippuric acid, may also contribute to cognitive decline in dialysis patients. Metagenomic studies suggest that key enzymes involved in the production of IS, PAG, and TMAO precursors are more common in the gut microbiota of patients with ESRD requiring HD (Zhang et al., 2023). TMAO is a gut-derived UT produced when dietary substrates such as choline and carnitine are metabolized by gut microbes to produce trimethylamine, followed by hepatic oxidation (Li J. et al., 2022). Especially when gut dysbiosis occurs, TMAO production and accumulation become more pronounced. A recent study suggested that gut dysbiosis in PD patients may increase TMAO production. TMAO may be linked to phenotypic transition and fibrotic changes in peritoneal mesothelial cells and fibroblasts under high-glucose conditions through the TGF-β1/Smad2/3 and Wnt/β-catenin pathways. Peritoneal fibrosis is involved in the development of ultrafiltration failure (UFF), which may impair the removal of fluid and uremic toxins by PD (Xie et al., 2026). In dialysis patients, TMAO is more likely to accumulate because of reduced renal clearance (Ribeiro et al., 2024). Epidemiological studies have suggested a positive association between circulating TMAO levels and the prevalence of cognitive impairment (Long et al., 2024). However, a prospective cohort study showed no association between plasma TMAO levels and cognitive function, neuroimaging markers, or incident dementia (Yaqub et al., 2024). Experimental studies have provided some mechanistic clues regarding the potential neurotoxicity of TMAO, but these data are mainly derived from animal models or *in vitro* experiments and cannot be directly extrapolated to dialysis patients. In a mouse model of vascular dementia, high circulating TMAO levels were reported to activate the NLRP3 inflammatory pathway and suppress hippocampal SIRT1 signaling, thereby exacerbating oxidative stress-induced neuroinflammation and apoptosis, which was associated with cognitive dysfunction (Deng et al., 2022). TMAO has also been shown to induce brain aging and age-related cognitive dysfunction in SAMR1 mice (Li et al., 2018). In addition, TMAO may disrupt the BBB by inhibiting endothelial TGF-β signaling and may activate inflammation and oxidative stress, thereby participating in cognitive dysfunction (Liu et al., 2026). However, direct evidence in dialysis patients remains insufficient. Therefore, TMAO is currently better regarded as a candidate marker or potential mediator of gut-derived metabolic disturbance rather than a definite causal factor. In contrast, PAG and hippuric acid has more direct clinical evidence in patients with CKD. In the CKD-REIN cohort, higher serum PAG levels were independently associated with cognitive impairment in patients with non-dialysis-dependent CKD (Levassort et al., 2025). Similarly, one HD study found that PAG and hippuric acid were markers of cognitive impairment (Kurella Tamura et al., 2016). In summary, metabolomic studies in dialysis patients suggest that TMAO, PAG, and hippuric acid are related to cognitive impairment. However, current evidence and mechanistic reproducibility remain limited, and larger longitudinal studies are still needed.

Third, bile acid dysregulation may represent another form of gut-derived metabolic reprogramming in CKD and dialysis patients. Multi-omics studies have shown that gut bacteria enriched in patients with ESRD encode and enrich functions related to bile acids (BAs) biosynthesis (Wang et al., 2020). Previous studies have suggested that gut microbiota dysbiosis can alter the bile acid profile and may take part in the development and progression of cognitive impairment-related diseases, such as Alzheimer’s disease and Parkinson’s disease (Collins et al., 2023; Mohanty et al., 2024). Under normal conditions, bile acids can enter the central nervous system by passive diffusion or transporter-mediated transport across the BBB (Butcher and Arthur, 2025). Animal and *in vitro* studies have shown that bile acid dysregulation, such as excessive accumulation of deoxycholic acid (DCA), chenodeoxycholic acid (CDCA), and hyodeoxycholic acid (HDCA), may damage BBB function. This process may involve Rac1-dependent occludin phosphorylation, which disrupts tight junctions in brain microvascular endothelial cells and increases BBB permeability (Quinn et al., 2014; Xing et al., 2023). In patients undergoing HD, targeted metabolomics studies have found marked changes in the circulating bile acid profile compared with healthy controls. These changes include decreased proportions of total unconjugated bile acids and SBAs, and increased proportions of total conjugated bile acids, primary bile acids (PBAs), taurine-conjugated bile acids, and glycine-conjugated bile acids (Li R. et al., 2019). Similar bile acid metabolic disturbances have also been observed in patients undergoing PD. Compared with healthy controls, the proportion of lithocholic acid (LCA) in plasma metabolites decreased to 37% in the peritoneal dialysis group, while the proportion of conjugated sulfated bile acids increased to 44% (Manokasemsan et al., 2025). However, direct evidence on the relationship between bile acids and cognitive impairment in dialysis patients is still limited. The CRIC cohort study showed that, in patients with CKD stages 2–4, higher circulating DCA levels were independently associated with cognitive impairment measured by the Category Fluency test (Mortaji et al., 2025). Overall, bile acid abnormalities may be a potential part of dialysis-related gut-derived metabolic reprogramming. However, studies that integrate bile acid profiles, gut microbiota, BBB injury markers, and longitudinal cognitive outcomes in dialysis patients are still lacking. Fourth, SCFAs deficiency and “leaky gut” are key links in metabolic and inflammatory amplification. SCFAs, including acetate, propionate, and butyrate, are primarily generated by beneficial gut bacteria through the fermentation of dietary fiber. They are considered to have potential kidney-protective effects (Li et al., 2023; Tian X. et al., 2023). They are also important metabolites for maintaining energy supply in colonic epithelial cells, tight junction stability, and immune tolerance. Multi-omics studies suggest that gut dysbiosis in the ESRD dialysis stage can systematically reshape the host metabolome and affect kidney failure-related phenotypes (Wang et al., 2020). Deep metagenomic evidence in ESRD shows that commensal bacteria such as *Prevotella* and *Roseburia* are often reduced in dialysis patients, and functional analysis suggests relatively impaired butyrate production (Zhang et al., 2023). Similarly, another study found that SCFAs-producing bacteria, such as *Roseburia*, *Faecalibacterium*, and *Coprococcus*, decreased with CKD progression (Wang H. et al., 2023). Butyrate is one of the SCFAs with relatively strong functional evidence. In dialysis patients, the reduction of SCFAs-producing bacteria and insufficient SCFAs production are common. A study in MHD patients suggested that HD patients often have gut dysbiosis caused by immune deficiency, and SCFAs-producing bacteria may be depleted under this condition (Lv et al., 2025). At the same time, increased UTs burden can inhibit SCFAs-producing bacteria and promote the growth of uremia-related bacterial taxa, thereby forming a vicious cycle of “increased UTs–decreased SCFAs” (Zhao et al., 2021b). These changes may weaken butyrate-mediated inhibition of histone deacetylase (HDAC), GPR109A/GPR43 signaling, and STING/NF-κB/p65 pathway-mediated NLRP3 pyroptosis. This may lead to kidney function decline, downregulation of tight junction proteins, gut barrier injury, and increased LPS burden. As a result, systemic inflammation may be amplified, BBB vulnerability and neuroinflammation may be promoted, and cognitive decline may finally be aggravated (Tian X. et al., 2023; Milovanova et al., 2026; Li H.-B. et al., 2022).

## 3 Potential mechanisms by which gut microbiota and their metabolites contribute to cognitive impairment in dialysis patients

### 3.1 Gut dysbiosis in dialysis patients may aggravate cognitive impairment

As CKD progresses, cognitive function gradually declines (Capasso et al., 2025). This decline is especially obvious in patients receiving long-term HD or PD (Zhang et al., 2024; Kalirao et al., 2011). Traditionally, the worsening of neurocognitive function in patients with CKD has been mainly attributed to vascular disease and traditional cardiovascular risk factors, uremic metabolites, and non-traditional kidney disease-related factors, such as depression, insomnia, and polypharmacy (Capasso et al., 2025; Karakizlis et al., 2022; Tian et al., 2021; Hafez et al., 2023). On this basis, dialysis itself may introduce additional risks. In HD patients, intermittent clearance of uremic toxins, hemodynamic stress, dialysis-related sodium fluctuations, and inflammatory stimulation may expose the brain to a combined risk of ischemia, inflammation, and toxin burden, which may be associated with cognitive impairment. In PD patients, long-term exposure to glucose- and glucose degradation product-containing dialysate, together with continuous peritoneal stimulation and peritoneal fibrosis, may also represent potential risk factors for cognitive decline (McIntyre and Jain, 2025). In the past few years, emerging evidence has indicated that gut dysbiosis may contribute to cognitive impairment among dialysis patients (Zhu et al., 2020; Wang J. et al., 2023; Martín-del-Campo et al., 2024). At the dialysis stage, several factors, including UTs retention, chronic inflammation, diet and drug exposure, and the dialysis process itself, may further disturb the gut microbial ecosystem. This dysbiosis is characterized by reduced beneficial commensal bacteria and SCFAs-producing bacteria, enrichment of pro-inflammatory or potential toxin-producing bacteria, and impaired microbial functional homeostasis. It is also accompanied by increased UTs, bile acid disorder, decreased SCFAs, and increased LPS burden (Li et al., 2024; Hsu et al., 2025; Zhao et al., 2025).

These microbial and metabolic alterations may be linked to cognitive function through several interconnected pathways, including processes related to BBB integrity, myelination, neuronal survival and proliferation, appetite, energy metabolism, thermoregulation, mood, anxiety, depression, stress responses, and local inflammation (Wagner et al., 2025). Animal and clinical studies further support this mechanistic link. In a CKD mouse model, UTs were shown to trigger potassium efflux in microglia, increase interleukin-1β (IL-1β) release, and induce IL-1β receptor 1 (IL-1R1)-mediated neurological dysfunction (Zimmermann et al., 2024). Another CKD animal study showed that elevated IS could disrupt the BBB by activating the AhR signaling and was associated with cognitive impairment (Bobot et al., 2020). Clinically, a prospective cohort study including 15 patients with ESRD showed that BBB permeability was higher in ESRD patients than in healthy controls. In this study, 78.6% of patients were considered to have cognitive impairment, with lower MoCA scores in memory, attention, and language domains, as well as higher Beck Depression Inventory scores (Bobot et al., 2024). In contrast, SCFAs may have protective effects on the BBB. Studies have shown that SCFAs can promote the acetylation of histone and non-histone proteins by inhibiting histone deacetylases, and may interact with transcription factor-related pathways, such as NF-κB and Nrf2, helping maintain BBB integrity (Fock and Parnova, 2023). Notably, not all gut-derived metabolites exert uniformly detrimental effects. For example, TMAO may enhance BBB integrity at physiological concentrations through the tight junction regulator annexin A1 and protect the BBB from inflammatory injury. However, its precursor trimethylamine (TMA) may impair BBB function and disrupt tight junction integrity (Hoyles et al., 2021). In addition, in C57BL/6 J mice, LPS was shown to induce neuroinflammation and cognitive impairment by activating NF-κB signaling and microglia (Zhao et al., 2019).

Taken together, through the gut–brain–kidney axis, dialysis-related gut dysbiosis and metabolic reprogramming may jointly promote gut barrier injury, amplification of systemic inflammation and oxidative stress, and subsequent BBB disruption, neuroinflammation, and cognitive decline (Zhu et al., 2025).

### 3.2 Mechanisms of the microbiota–gut–brain–kidney axis in cognitive impairment in dialysis patients

Dialysis-related cognitive decline is not the result of injury to a single organ. It is more likely a multi-organ network process caused by long-term interactions among the kidney, gut, brain, and neuroendocrine system. The microbiota–gut–brain–kidney axis proposed in recent years provides an integrated framework for understanding this complex mechanism (Yang et al., 2018). In this framework, the gut microbiota and its metabolites are key hubs (Martín-del-Campo et al., 2024). Through bidirectional interactions with the gut barrier, immune and inflammatory responses, the autonomic nervous system, and neuroendocrine pathways, they link kidney function decline, systemic inflammation, and brain dysfunction (Zhu et al., 2025; Yang et al., 2018). After patients enter the dialysis stage, UT retention, chronic low-grade inflammation, hemodynamic fluctuations, dietary restriction, and drug exposure may further amplify this network imbalance. As a result, cognitive decline may show continuous progression and self-reinforcing features (Wang J. et al., 2023; Martín-del-Campo et al., 2024; Wagner et al., 2025).

Vascular endothelial dysfunction is common in dialysis patients (Capasso et al., 2025). It may promote neurodegenerative diseases and cognitive decline (Han et al., 2020). Clinical studies also support this mechanistic pathway. In HD patients, several endothelial-related biomarkers, including angiopoietin-2 (ANGPT2), intercellular adhesion molecule-1 (ICAM-1), and syndecan-1, are significantly associated with baseline cognitive function (Freire De Medeiros et al., 2020). Another study in MHD patients further showed that syndecan-1 was independently associated with accelerated cognitive decline and had good predictive ability for severe cognitive impairment (Libório et al., 2024). At the dialysis stage, gut metabolism shifts toward reduced carbohydrate fermentation and increased generation of gut-derived UT precursors. This leads to increased toxic metabolites, such as IS, PCS, Hcy, TMAO, LPS, and SBAs, and decreased protective metabolites, such as SCFAs (Laiola et al., 2025; Li et al., 2024; Krukowski et al., 2026). These changes may promote a vascular microenvironment characterized by increased oxidative stress, reduced nitric oxide (NO) bioavailability, and pro-inflammatory activation. This may damage the gut barrier, allow related toxic metabolites to enter the blood, impair the BBB and neurovascular function, and finally promote cognitive decline (Harlacher et al., 2022). At the same time, tryptophan metabolism is reprogrammed by the gut microbiota in dialysis patients (Zhang et al., 2023; Kuo et al., 2025). In a CKD rat model, gut microbial tryptophan metabolism shifted toward the kynurenine pathway. This was accompanied by increased IS, PCS, and kynurenine, together with impairments in cognitive domains such as learning and memory (Ibos et al., 2024). According to current evidence, IS has stronger clinical and mechanistic support than PCS in cognitive impairment. A clinical case–control study showed that ESRD patients with cognitive impairment had significantly higher IS levels than healthy controls (Hsieh et al., 2025). In a study of IS and PCS in HD patients, circulating free IS, but not PCS, was associated with lower cognitive test scores (Lin et al., 2019b). Mechanistically, IS may affect BBB integrity by activating the AhR, and it may be linked to inflammatory injury. In contrast, evidence for PCS in cognitive impairment mainly comes from animal studies. For example, PCS was associated with neuronal injury, apoptosis, oxidative stress, and increased neuroinflammation in nephrectomized mice (Sun et al., 2020). Therefore, IS currently has more direct evidence in the AhR–BBB–cognition pathway, whereas PCS may be involved in brain dysfunction mainly through indirect pathways, such as neuroinflammation, oxidative stress, and vascular injury.

Accumulating studies suggest that gut-derived UTs can damage BBB integrity and lead to cognitive impairment. This has been reported in both ESRD/dialysis animal models and patients (Bobot et al., 2024; Bobot et al., 2020). LPS and gut-derived UTs, such as IS and IAA, can impair BBB function through signaling pathways such as TLR4 and AhR (Wu P.-H. et al., 2020; Wu P.-H. et al., 2024; Wei et al., 2024). TMAO can increase BBB permeability by reducing tight junction proteins in microvessels (Hernandez et al., 2022). After BBB permeability increases, peripheral inflammatory factors, LPS, SBAs, and gut-derived UTs, especially PBUT, are more likely to enter the central nervous system (CNS) or increase brain exposure. This may amplify neuroinflammation and oxidative stress in the CNS. After these abnormal molecules enter the CNS, they may further activate microglia and astrocytes. They can increase the release of pro-inflammatory cytokines, such as interleukin-1β (IL-1β), IL-6, and tumor necrosis factor-*α* (TNF-α), and form a sustained inflammatory amplification effect through related signaling pathways, including TLR4 and NF-κB/AhR. For example, LPS can aggravate cognitive impairment through the TLR4-mediated inflammatory signaling pathway (Wei et al., 2025). IS can increase oxidative stress and glial inflammation via pathways like AhR/NF-κB, thereby worsening neuronal injury (Adesso et al., 2017). Chronic psychological and physiological stress is common in patients with advanced CKD and in those receiving maintenance dialysis. Growing evidence suggests that this condition is accompanied by dysfunction of the hypothalamic–pituitary–adrenal (HPA) axis (Boswell et al., 2024; Sagmeister et al., 2023). Sustained exposure to high levels of glucocorticoids and circadian rhythm disruption can impair hippocampal and prefrontal cortex function. They can also inhibit neurogenesis and synaptic plasticity and increase oxidative stress burden, thereby increasing susceptibility to cognitive decline (Zahedi et al., 2025; Gulyaeva, 2023).

**Figure 1:**
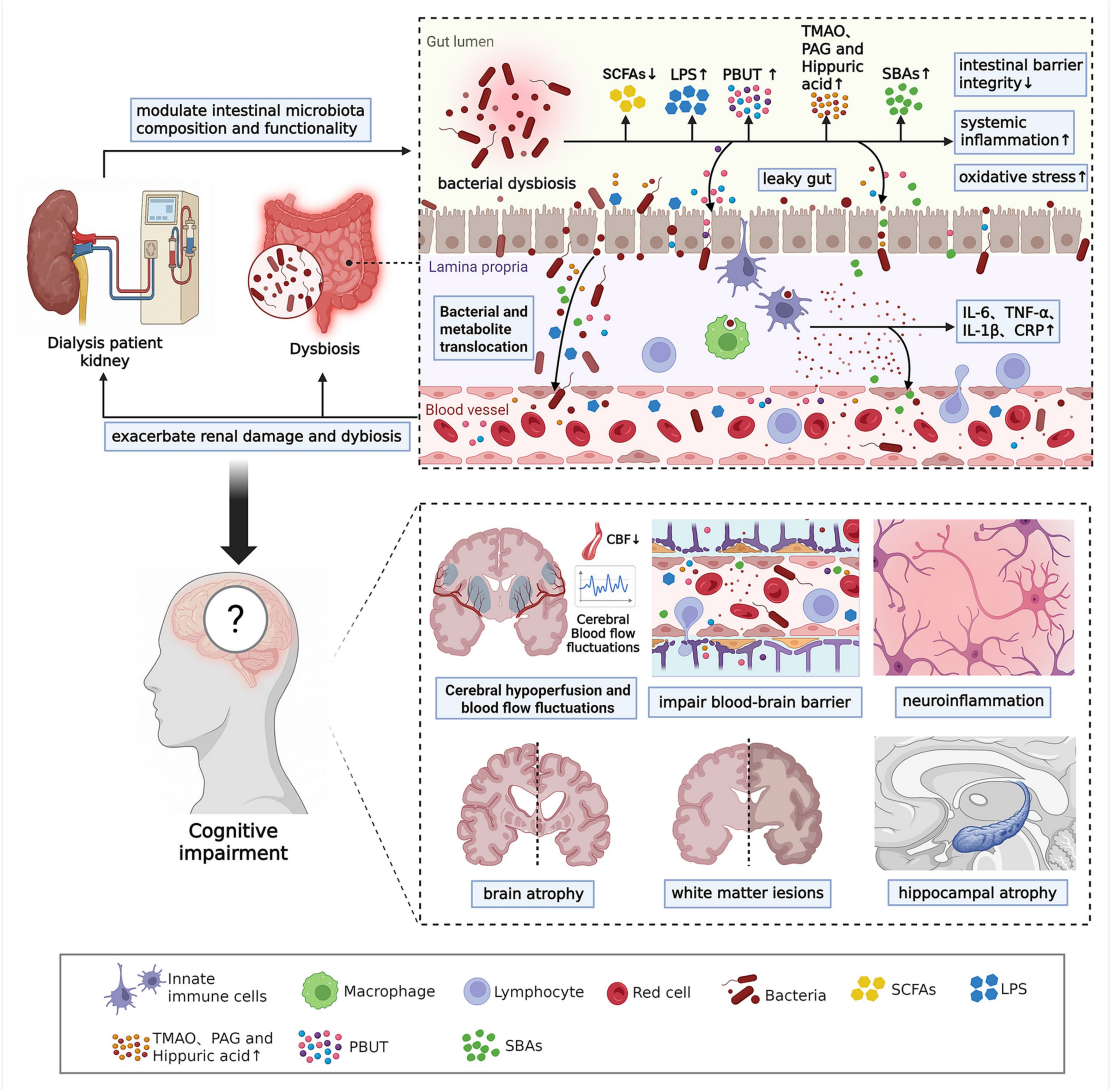
Mechanisms of cognitive dysfunction in dialysis patients. Dialysis patients commonly exhibit gut microbiota dysbiosis, which may further aggravate renal injury and worsen intestinal microecology, forming a vicious gut–kidney cycle. Gut microbiota dysbiosis can lead to decreased SCFAs and increased production and accumulation of gut-derived metabolites or inflammation-related molecules, including LPS, PBUT, TMAO, PAG, hippuric acid, and SBAs. After intestinal barrier integrity is impaired, bacteria and their metabolites can translocate into the circulation, inducing systemic inflammation and oxidative stress, accompanied by elevated IL-6, TNF-α, IL-1β, and CRP. These circulating inflammatory mediators and uremic toxins, together with dialysis-related cerebral hypoperfusion and CBF fluctuations, may further damage the BBB, induce neuroinflammation, and promote brain atrophy, white matter lesions, and hippocampal atrophy, ultimately contributing to the onset and progression of cognitive impairment. SCFAs, short-chain fatty acids; LPS, lipopolysaccharide; PBUT, protein-bound uremic toxins; TMAO, trimethylamine N-oxide; PAG, phenylacetylglutamine; SBAs, secondary bile acids; IL-6, interleukin-6; TNF-α, tumor necrosis factor-α; IL-1β, interleukin-1β; CRP, C-reactive protein; CBF, cerebral blood flow; BBB, blood–brain barrier.

In summary, kidney function decline leads to the accumulation of UTs, damages the gut barrier, and changes the composition of the gut microbiota. Gut dysbiosis can reduce SCFAs-producing bacteria and increase LPS burden. As a consequence, intestinal barrier integrity may be further disrupted, leading to increased permeability, microbial product translocation, and enhanced systemic inflammatory responses. At the same time, remodeling of microbial metabolic function can increase the production and accumulation of gut-derived UTs, such as IS, PCS, Hcy, and TMAO. These abnormal metabolites may contribute to brain dysfunction by disrupting the BBB and promoting neuroinflammation, thereby aggravating cognitive decline. In addition, chronic stress in dialysis patients can activate the HPA axis, and cortisol dysregulation may further worsen cognitive injury and immune imbalance. Brain dysfunction may also be associated with kidney function decline through overactivation of the sympathetic nervous system (SNS) and the renin–angiotensin–aldosterone system (RAAS). Specifically, dialysis-associated cognitive impairment involves interactions among the gut–kidney axis, gut–brain axis, brain–kidney axis, and HPA axis, forming a vicious network that promotes cognitive decline (Zhu et al., 2025; Wagner et al., 2025) (Figure 1).

## 4 Gut microbiota-targeted intervention strategies for cognitive impairment in dialysis patients

### 4.1 Lifestyle and dietary patterns

Many dialysis patients face risks such as hyperkalemia, hyperphosphatemia, chronic metabolic acidosis, bone deterioration, abnormal blood pressure, and edema (Natale et al., 2021; Karaboyas et al., 2021; Hu L. et al., 2022; Korus et al., 2025). Therefore, dietary intervention should limit potassium, phosphorus, sodium, and fluid intake while ensuring adequate protein and essential nutrients (Rastogi et al., 2021; Naber and Purohit, 2021). According to the updated National Kidney Foundation Kidney Disease Outcomes Quality Initiative (KDOQI) clinical practice guideline on nutrition in CKD, dietary protein restriction may be an effective strategy for delaying the progression of CKD (Ikizler et al., 2020). However, related studies suggest that its kidney-protective effect may be limited (Amiri Khosroshahi et al., 2025). After patients enter the dialysis stage, more attention should be paid to protein-energy wasting (PEW). Under the premise of meeting the minimum protein requirement, the structure of protein sources should be optimized, and dietary fiber intake should be increased. This may help maintain nutritional status and support cognitive protection (Kanbay et al., 2025). Evidence suggests that plant-based dietary patterns, such as the Mediterranean diet and other plant-dominant diets, may help improve gut microbiota diversity and restore beneficial gut bacteria (Merra et al., 2020; Marrone et al., 2025). In HD patients, plant-based diets and food choices may affect the levels of gut-derived UTs, including IS and PCS, as well as gut microbiota characteristics (Stanford et al., 2021). The plant-dominant low-protein diet (PLADO) emphasizes the preferential replacement of animal protein with plant protein. Its core idea is to optimize the structure of protein sources, rather than to further reduce total protein intake during the dialysis stage (Marrone et al., 2025). Mechanistically, Plant-based dietary patterns are generally linked to greater gut microbial *α* diversity and a higher abundance of beneficial commensal bacteria. These dietary patterns may increase the production of antioxidant and anti-inflammatory metabolites, such as SCFAs and indole-3-propionic acid (IPA). They may also reduce LPS burden and pro-inflammatory metabolic output. These effects may help reduce oxidative stress and chronic low-grade inflammation, and improve immune homeostasis and barrier function (Kalantar-Zadeh et al., 2020; Miao et al., 2022; Shen et al., 2024; Konopelski and Mogilnicka, 2022). Dietary fiber intervention can reshape the structure of the gut microbiota (Vinelli et al., 2022). Clinical evidence also suggests that fiber supplementation generally helps reduce gut-derived UTs and inflammatory burden (Wathanavasin et al., 2025). However, some studies did not observe a parallel increase in SCFAs, suggesting that the metabolic response may be delayed (Oliver et al., 2021). In addition to diet, exercise is an important non-pharmacological strategy for cognitive protection in dialysis patients. Studies have shown that intradialytic exercise training can slow the progression of cognitive impairment (Mavrommatis et al., 2025). Clinical evidence from randomized controlled and multicenter studies further suggests that exercise combined with cognitive training may confer benefits for alertness and physical function (Bogataj et al., 2024; Bogataj et al., 2023; Ghildayal et al., 2025). These effects may be mediated by the brain-derived neurotrophic factor (BDNF)-related “muscle–brain–kidney axis” and antioxidant and anti-inflammatory effects (Deus et al., 2021). Elastic band resistance training may have greater value for wider use because it is safe and easy to perform (Feng et al., 2025). Overall, optimizing dietary structure and maintaining regular exercise may jointly support cognitive function in dialysis patients through several pathways. These include improving gut microecology, reducing gut-derived UT burden, relieving inflammation and oxidative stress, and supporting neuroplasticity. Therefore, lifestyle-based microbiota intervention may be a feasible direction for cognitive protection in dialysis patients.

### 4.2 Probiotics and microbiota-based preparations

Probiotics, prebiotics, and synbiotics are three commonly used microbiota-based preparations. Probiotics are defined as live microorganisms that, when administered in adequate amounts, confer beneficial effects on host health (Hill et al., 2014). Prebiotics are dietary substrates that support host health by selectively stimulating the activity or growth of beneficial microorganisms (Gibson et al., 2017). Synbiotics are combined preparations of probiotics and prebiotics. In recent years, these interventions have been used in studies on gut microbiota modulation in CKD and dialysis patients. They aim to regulate the structure and function of the gut microbiota, reduce the production of protein fermentation-related gut-derived UTs, such as IS and PCS, and improve gut barrier function and inflammatory/oxidative stress status. Therefore, they may provide an intervention target for the metabolic–immune pathways related to dialysis-associated cognitive impairment in the gut–brain–kidney axis (Huang and Chen, 2024). Current evidence suggests that probiotic, prebiotic, and synbiotic interventions vary greatly in study populations, preparation components, doses, treatment duration, and outcome indicators. These differences lead to inconsistent overall effects and low certainty of evidence. Their real clinical benefits still need to be confirmed by high-quality trials (Cooper et al., 2023). For example, one randomized controlled trial (RCT) showed that probiotics, prebiotics, and synbiotics may reduce some gut-derived UTs, including total/free IS and PCS. However, differences in preparation type, strain, dose, treatment duration, and patient population may lead to inconsistent conclusions (Cedillo-Flores et al., 2025). It should be emphasized that a recent metagenomic study in PD patients highlighted the importance of a plant-based diet. This study found that lower estimated glomerular filtration rate (eGFR) in PD patients was associated with increased UTs, such as PCS and indoles, and a decreased ratio of carbohydrate-active enzymes (CAZymes) related to plant-derived polysaccharide degradation compared with those related to animal-derived glycan degradation. This finding suggests insufficient fermentable dietary substrates and reflects inadequate fiber intake in dialysis patients (Krukowski et al., 2026).

In terms of mechanistic evidence, a mouse model of CKD first showed that *Faecalibacterium prausnitzii* (*F. prausnitzii*), as a commensal bacterium, could reduce UTs production, regulate gut dysbiosis, and alleviate kidney inflammation through the butyrate–GPR43 axis (Li H.-B. et al., 2022). Animal studies have shown that synbiotics can improve gut dysbiosis and reduce fecal IS burden (Yang et al., 2021). A preclinical study using an Alzheimer’s disease model demonstrated that treatment with a mixture of probiotics could improve neuronal injury, A*β* and Tau pathology, and neuroinflammation by regulating phosphorylation in the AKT/GSK-3β pathway. This finally relieved cognitive impairment and pathological changes in mice (Xiao-hang et al., 2024). However, it should be noted that human evidence for cognitive benefits mainly comes from non-dialysis-related studies (Aljumaah et al., 2022). A clinical study involving patients with non-dialysis CKD stages 3–5 demonstrated that β-glucan prebiotic reduced plasma UTs, such as PCS, IS, and p-cresyl glucuronide (PCG), and restored normal *Prevotella* abundance, but it did not significantly improve kidney function (Ebrahim et al., 2022). Another study showed that a synbiotic preparation, NATUREN G^®^, reduced small intestinal permeability and plasma PCS and IS levels in non-dialysis patients (Cosola et al., 2021). Similar findings have also been observed in dialysis patients. For example, in MHD patients, 8-week dietary supplementation with high-amylose resistant starch reduced serum PCS levels, but IS levels did not change (Khosroshahi et al., 2019). It is worth noting that one study showed that inulin-type fructan prebiotic was not sufficient to reduce plasma TMAO levels in PD patients (Xiong et al., 2023). In addition, a study in PD patients suggested that prebiotics may decrease serum uric acid levels and support the preservation of residual kidney function in dialysis patients (He et al., 2022). A clinical study in HD patients showed that p-inulin prebiotic significantly promoted the growth of SCFA-producing bacteria, such as Clostridiales. However, this study did not find that prebiotics reduced UTs concentrations, such as IS, PCS, and TMAO (Raj et al., 2021). After synbiotic supplementation in HD patients, serum IS and PCS levels decreased, while IL-6 and malondialdehyde (MDA) were also reduced. A randomized controlled study also found that in HD patients, synbiotic treatment combined with DVB-PVP hemodialysis can simultaneously reduce both pre-dialysis and post-dialysis levels of IS and PCS (Rocchetti et al., 2020). These findings suggest that synbiotics may reduce gut-derived UTs burden and may also relieve chronic inflammation and oxidative stress (Kuskunov et al., 2023).

Overall, probiotic, prebiotic, and synbiotic interventions may help reduce serum IS, PCS, LPS, and pro-inflammatory mediators, including IL-6, TNF-*α*, IL-1β, and C-reactive protein (CRP), in dialysis patients. These effects may improve systemic inflammation and oxidative stress, repair the gut barrier, and reduce gut permeability and bacterial translocation (Nguyen et al., 2021). These benefits may further reduce the inflammatory burden of the neurovascular unit, improve BBB vulnerability and neuroinflammation, and provide a potential mechanistic basis for relieving or delaying dialysis-associated cognitive impairment.

### 4.3 Fecal microbiota transplantation (FMT)

FMT is an intervention strategy in which processed fecal microbiota from healthy donors are transferred into individuals with gut dysbiosis to rebuild microbial homeostasis. Research indicates that the use of FMT has been extensively explored in inflammatory bowel disease, metabolic syndrome, and conditions related to the gut-brain axis, such as depression and Alzheimer’s disease (Qiu B. et al., 2023; Yi et al., 2025). Recent studies show that FMT may reduce inflammatory burden, improve barrier function, and regulate neuroimmune responses by reshaping gut microbial composition and metabolic output. Therefore, it may become a potential strategy for improving cognitive function (Alaeddin et al., 2025). However, direct clinical evidence for FMT in dialysis-associated cognitive impairment remains very limited (Cymbal et al., 2025). In CKD, the available human evidence mainly comes from small-scale studies that focus on renal or microbiota-related outcomes rather than cognitive endpoints. Notably, a single-center, double-blind, randomized, placebo-controlled clinical trial evaluated FMT in 28 patients with stage 2–4 CKD associated with diabetes and hypertension. Compared with those in the placebo group, the FMT group showed decreased relative abundance of *Firmicutes* and *Actinobacteria*, and increased relative abundance of *Bacteroidetes* and *Proteobacteria*. This suggests that gut microecology can be reshaped to some extent and may be related to slower CKD progression. However, 30 and 90 days following therapy, the abundance of *Roseburia* decreased in CKD patients, and this change may be accompanied by increased blood glucose and CRP levels during the same period (Arteaga-Muller et al., 2024; Zhao et al., 2021a; Jiang et al., 2016). A multicenter retrospective cohort study involving 372 patients treated with FMT showed that FMT had favorable efficacy and safety for the treatment of *Clostridioides difficile* infection in children and young adults (Nicholson et al., 2020). In addition, a case report showed that FMT could be used to treat recurrent *Clostridioides difficile* infection in a patient receiving PD (Marasco et al., 2025). Another study involving three children receiving kidney replacement therapy also found that FMT had favorable therapeutic effects on recurrent *C. difficile* infection in this population (Samaey et al., 2024). These findings suggest that FMT may be feasible for modulating gut microecology in selected dialysis or kidney replacement therapy populations. However, these studies did not assess cognitive outcomes in dialysis patients. Therefore, their findings can only provide indirect support for the feasibility of microbiota remodeling in CKD, rather than direct evidence for FMT in DACI.

Overall, FMT may reduce neuroinflammation and BBB vulnerability at the level of the gut–brain–kidney axis. It may act by continuously correcting gut dysbiosis, reducing LPS translocation and UTs precursor production, relieving systemic inflammation and oxidative stress, and inhibiting kidney fibrosis. These effects provide a theoretical basis for improving or delaying dialysis-associated cognitive impairment. However, its efficacy and safety in dialysis patients still need further validation. Important issues include infection risk, immune status, drug exposure, differences in dialysis modality, and the optimal route and duration of treatment. More high-quality clinical studies with cognitive outcomes as endpoints are needed.

### 4.4 Targeted clearance strategies for uremic toxins and their derivatives

Accumulating evidence indicates that gut microbiota-derived, BAs, PBUT, including IS, PCS, and IAA, together with Hcy and arginine-derived guanidino compounds (GCs), such as guanidinosuccinic acid, guanidine, and methylguanidine, are considered major UTs that may cause brain injury in dialysis patients (Wu P.-H. et al., 2024; Natale et al., 2021; Jiang et al., 2024; Shah et al., 2024). Detoxification strategies targeting these metabolites are now an important approach for relieving dialysis-associated neurological injury and improving cognitive outcomes (McIntyre and Jain, 2025; Watanabe et al., 2021).

The oral spherical carbon adsorbent AST-120 can physically adsorb indole and its precursors in the gut. It reduces the indole-derived burden and decreases IS production at the source. Therefore, AST-120 is regarded as one of the representative gut-derived UT-lowering strategies (Andrews et al., 2025; Cha et al., 2022). A network meta-analysis in CKD patients suggested that moderate-dose AST-120, approximately 6 g/day, may reduce composite renal outcomes and the risk of progression to ESKD (Su et al., 2021). Clinical studies further suggest that moderate-dose AST-120 may reduce IS burden in CKD or HD patients, although its direct cognitive benefit in dialysis patients remains uncertain. A study of 37 ESKD patients receiving HD showed that discontinuation of AST-120 at 6 g/day was followed by a marked but highly variable increase in both free and protein-bound IS within 7 days. The dialysis clearance of IS was also influenced by serum albumin levels. These findings suggest that nutritional status and protein-binding properties may affect PBUT removal (Yoshida et al., 2026). Another multicenter randomized open-label trial involving 96 HD patients showed that AST-120 at 6 g/day for 4 weeks was associated with lower IS, PCS, and TNF-*α* levels, as well as reduced uremic pruritus severity (Wu C.-C. et al., 2024). In addition, a 48-week randomized, open-label, multicenter, parallel-group trial involving 150 pre-dialysis CKD patients showed that AST-120 treatment was associated with reduced serum IS levels and improved cognitive function. IS levels were also positively correlated with the severity of cognitive impairment (Cha et al., 2022). However, because this study enrolled pre-dialysis CKD patients, its relevance to cognitive benefit in dialysis patients remains indirect. A systematic review also suggested that AST-120 supplementation in HD patients may be associated with relatively favorable effects in longitudinal studies. However, this finding should be interpreted cautiously because of heterogeneity in study design and outcome measures (Saar-Kovrov et al., 2021). Animal studies provide mechanistic support for the potential neuroprotective role of AST-120. In CKD animal models, dietary intervention with 10% AST-120 for 8 weeks was associated with significantly lower IS levels in both serum and the hippocampus (Li et al., 2021). In 5/6 nephrectomized CKD rats, daily administration of 8% AST-120 at 2.04 g/kg was associated with improvement in cognitive and anxiety-like behaviors. These changes were accompanied by improved electrophysiological parameters, including local field potentials and excitatory postsynaptic potentials, as well as reduced AQP4/GFAP co-expression in the hippocampus. These findings suggest partial improvement in spatial learning and memory impairment (Yu et al., 2024). Another animal study showed that oral AST-120 was associated with reduced plasma IS levels in 5/6 nephrectomized rats (Sivertsson et al., 2023). A meta-analysis of CKD animal models also showed that AST-120 treatment was associated with a significant reduction in IS levels (Altunkaynak et al., 2024). Mechanistically, AST-120 may reduce peripheral IS burden, alleviate vascular endothelial injury, and preserve BBB integrity. This may limit the entry of UTs into the CNS (Bobot et al., 2020). CKD mouse studies further showed that AST-120 was associated with lower IS accumulation and reduced NLRP3 inflammasome-related neuroinflammation in the frontal cortex and hippocampus. These changes were reflected by reduced microglial activation and lower IL-1β and IL-18 levels, together with improvement in spatial memory impairment (Li et al., 2021). Recent electrophysiological studies also suggest that improvement of the inflammatory microenvironment may help restore synaptic transmission efficiency and improve spatial memory impairment (Natale et al., 2021). Therefore, current animal evidence suggests that AST-120 may be involved in the regulation of CKD-related cognitive impairment through the pathway of “IS reduction–BBB protection–suppression of glial inflammatory responses–improvement of synaptic function.” However, its direct cognitive benefit in dialysis patients still requires further validation. In addition to conventional oral adsorbents, novel AST-120-related adsorption materials have also been explored. An *in vitro* materials study showed that EVOH nanofibers combined with AST-120 could effectively adsorb IS from blood and reduce IS concentrations under simulated blood circulation conditions (Sasaki et al., 2024). However, this evidence remains limited to in vitro adsorption and hemocompatibility evaluation. Its efficacy and safety in dialysis patients remain unverified. Clinical studies also suggest that oral AST-120 may partially improve gut microbiota structure in patients with advanced CKD. It was associated with increased SCFA-producing bacteria, including *Clostridium_sensu_stricto_1*, *Ruminococcus_2*, *Eubacterium_nodatum*, and *Phascolarctobacterium*. It was also associated with higher serum acetate and ursodeoxycholic acid (UDCA) levels, as well as lower PCS levels (Hsu et al., 2022). In addition, a retrospective study showed that AST-120 use was associated with lower risks of ESKD development and mortality (Lee et al., 2024). Overall, AST-120 has a relatively clear effect on reducing IS levels. It also shows potential in reducing neuroinflammation and improving cognitive behaviors in animal models. However, current evidence mainly comes from non-dialysis CKD patients, animal experiments, *in vitro* materials studies, or clinical studies using toxin reduction as the main endpoint. Future studies should further clarify the optimal dose, treatment duration, adherence, safety, and effects on standardized cognitive outcomes in maintenance dialysis patients.

Evidence from an early adenine-induced renal failure mouse model indicated that lubiprostone may reduce gut-derived UTs accumulation, including IS and hippuric acid, by reshaping the gut microbiota and improving the intestinal milieu. This showed a protective effect against CKD progression (Mishima et al., 2015). A deeper mechanism may involve regulation of intestinal epithelial function through the EP4 receptor–cyclic adenosine monophosphate (cAMP) pathway and improvement of barrier integrity, such as restoration of tight junction-related protein or gene expression, including Occludin. This may limit the intestinal translocation of bacterial products and toxins (Oak et al., 2022; Safari et al., 2023). However, a recent randomized phase II clinical trial found that, while delaying kidney function decline, lubiprostone significantly increased circulating levels of neuroprotective metabolites, such as spermidine (Watanabe et al., 2025). This metabolic improvement driven by gut microenvironment remodeling may effectively repair BBB integrity under uremic conditions by inhibiting mitochondrial oxidative stress in hippocampal microvascular endothelial cells, thereby reversing cognitive decline (Bobot et al., 2020).

### 4.5 Extracorporeal blood purification strategies beyond conventional HD and PD

Traditional HD mainly removes small solutes through diffusion (Jonny and Teressa, 2023). Previous clinical studies showed that increasing the frequency of HD alone did not improve executive function or global cognitive function (Kurella Tamura et al., 2013). Cooler dialysate HD also showed limited benefit for cognitive function (Dasgupta et al., 2024). Hemodiafiltration (HDF) introduces convective clearance in addition to diffusion (Lin and Silberzweig, 2026). Compared with HD, HDF can enhance the removal of some middle-molecular-weight toxins (Lima et al., 2022). HDF has also been associated with a lower risk of death in patients with ESRD compared with conventional HD (Vernooij et al., 2024; Peters et al., 2016). However, the removal of PBUT remains limited even with HDF. This is mainly because IS, PCS, and other PBUT are highly bound to albumin, and only the free fraction can be effectively transferred across the dialysis membrane. To overcome the limited removal of PBUT, several intensified blood purification strategies have been explored in recent years.

First, disruption of PBUT–albumin binding may increase the free toxin fraction and improve dialytic clearance. For example, in a clinical intervention study involving 18 maintenance HD patients, ibuprofen was infused into the arterial blood line during HD. Because ibuprofen competes with IS and PCS for albumin-binding sites, this intervention significantly increased the dialytic removal of IS and PCS and reduced their serum levels (Madero et al., 2019). Second, PBUT removal may also be improved by adsorptive blood purification. Hemoadsorption (HA) or hemoperfusion (HP) is an adsorption-based extracorporeal blood purification technique. During this process, blood passes through a perfusion or adsorption cartridge containing sorbents such as activated charcoal or resin, allowing target solutes to directly contact and bind to the adsorbent material (Ronco and Bellomo, 2022). New membrane materials have also been used to improve the removal of middle-molecular-weight toxins and some PBUT, including medium cut-off (MCO) membranes (Zhang et al., 2022) and mixed-matrix membranes (MMMs) with selective adsorption properties (Rodrigues and Faria, 2023). In addition, new adsorbent materials have been tested. For example, DVB-PVP resin removed approximately 56% of PCS and 54% of IS after 6 h of *in vitro* perfusion. However, in the *in vivo* study, the DVB-PVP cartridge showed an adsorption effect only for plasma IS (Rocchetti et al., 2020). Moreover, hemoadsorption combined with hemodialysis (HAHD) using a pHA130 adsorption cartridge showed stronger removal of IS, PCS, and other PBUT than HDF performed once every 2 weeks, regardless of whether HAHD was performed once or twice weekly (Yu et al., 2025).

Notably, combined blood purification strategies may further improve PBUT removal and may be associated with clinical benefit. A single-center, open-label trial in 30 maintenance HD patients found that HA + HD showed stronger removal of IS, PCS, and other PBUT than HD alone (Yu et al., 2025). In addition, a recent randomized, open-label, multicenter trial enrolled 1,362 maintenance HD patients with ESKD. Compared with HD alone, HA + HD was associated with lower all-cause mortality, cardiovascular mortality, and major adverse cardiovascular events (Lu W. et al., 2026). Beyond PBUT, HP or HA combined with HD has also shown interventional evidence for middle-molecular-weight toxin removal and clinical outcomes. A multicenter randomized controlled trial involving 438 ESRD patients showed that long-term HP + HD treatment, performed at least once weekly for 12 months, reduced β2-microglobulin (β2M) and parathyroid hormone (PTH) levels more effectively than HD alone. These findings suggest that HP + HD may have clinical value in improving the removal of middle-molecular-weight uremic toxins (Zhao et al., 2022). Another recent multicenter randomized controlled trial involving 135 maintenance HD patients compared the efficacy of different blood purification regimens. In this study, the HD + HDF + HP regimen, also referred to as triple dialysis, consisted of two HD sessions per week, one HDF session per week, and one additional combined HD + HP session per month. Compared with HD alone, defined as two HD sessions per week, and HD + HDF, defined as two HD sessions plus one HDF session per week, the HD + HDF + HP regimen further reduced the concentrations of small- and middle-molecular-weight uremic toxins. It also significantly reduced some PBUTs, especially Hcy and IS. In addition, this triple dialysis strategy was associated with slower cognitive decline and improved neurological outcomes in maintenance HD patients (Wang et al., 2025). Overall, current evidence suggests that intensified extracorporeal blood purification strategies have mechanistic plausibility and preliminary interventional support for enhancing PBUT or middle-molecular-weight toxin removal. However, whether these strategies can improve dialysis-associated cognitive impairment remains uncertain. Large randomized controlled trials with cognitive outcomes as primary endpoints are still needed.

**Figure 2:**
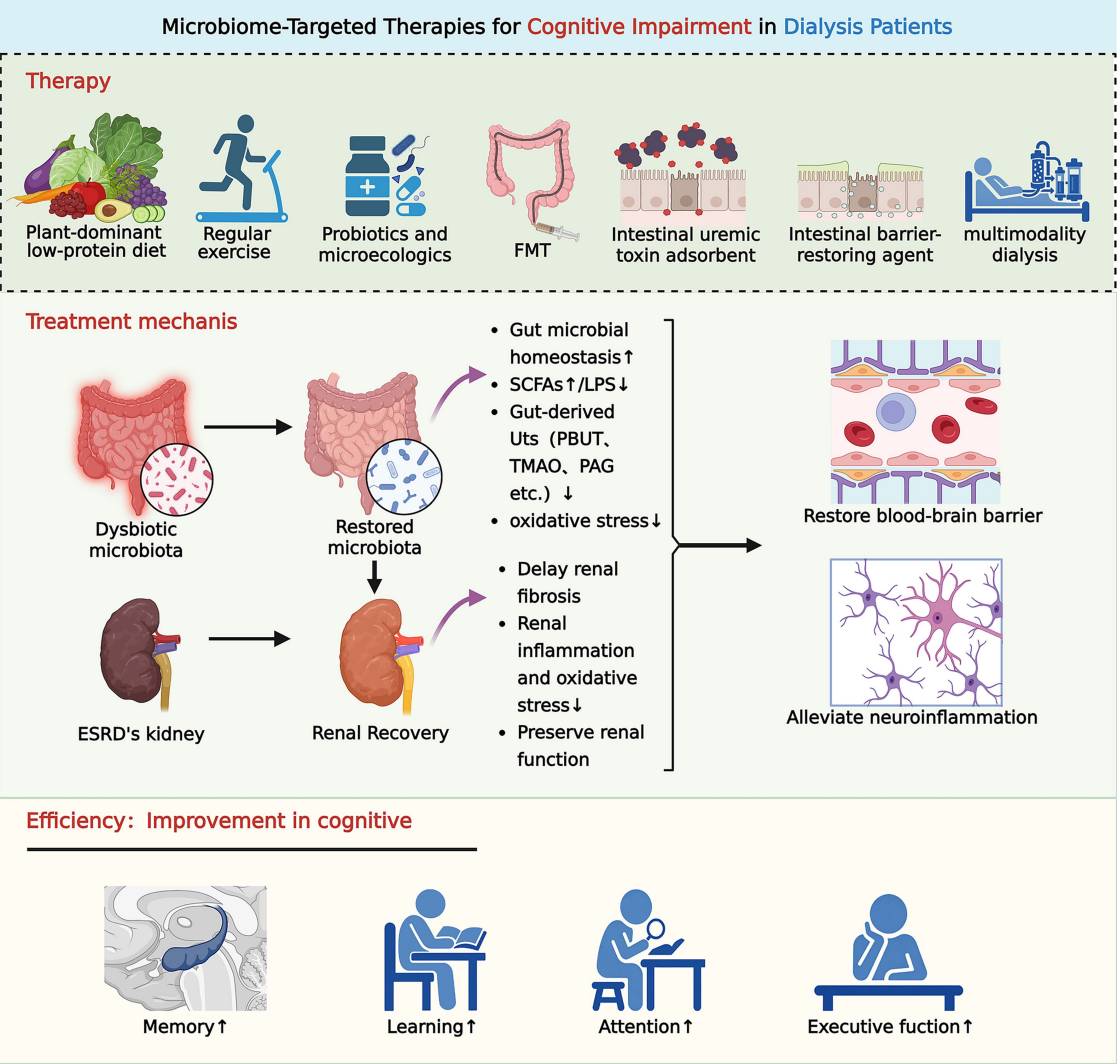
Microbiome-targeted therapies for cognitive impairment in dialysis patients. Plant-dominant low-protein diet, regular exercise, probiotics and microecologics, FMT, intestinal uremic toxin adsorbent, intestinal barrier-restoring agent, and multimodality dialysis may modulate the gut microecology of dialysis patients and promote the transition from dysbiotic microbiota to restored microbiota. Restoration of gut microbial homeostasis may increase SCFAs, decrease LPS, reduce the production and accumulation of gut-derived UTs, including PBUT, TMAO, PAG, etc., and alleviate oxidative stress. Meanwhile, these interventions may delay renal fibrosis, reduce renal inflammation and oxidative stress, and preserve renal function. By improving abnormalities in the gut–kidney axis and gut–brain axis, these therapies may further restore the BBB and alleviate neuroinflammation, ultimately improving cognitive performance, including memory, learning, attention, and executive function. SCFAs, short-chain fatty acids; LPS, lipopolysaccharide; UTs, uremic toxins; PBUT, protein-bound uremic toxins; TMAO, trimethylamine N-oxide; PAG, phenylacetylglutamine; BBB, blood–brain barrier.

Unlike HD, which can be combined with other extracorporeal blood purification strategies, approaches to reduce PBUT burden in PD patients mainly depend on optimization of the PD prescription, preservation of residual kidney function, and reduction of gut-derived toxin generation. During long-term PD, structural and functional alterations of the peritoneum may occur, including peritoneal fibrosis, which may affect ultrafiltration and solute removal (Shi et al., 2025). Recent studies also suggest that gut dysbiosis may be linked to PD-associated peritoneal fibrosis (Xie et al., 2026). PD can remove the free fraction of PBUT through transperitoneal diffusion. However, IS and PCS are highly bound to albumin, and their peritoneal clearance is therefore low. Earlier studies showed that the peritoneal clearance of PBUT such as IS and PCS was only approximately 0.3 ± 0.1 mL/min, which was much lower than that of water-soluble uremic toxins such as urea (Huang et al., 2012). A recent 5-year follow-up study further showed that the peritoneal clearances of small water-soluble solutes, including urea, creatinine, uric acid, and phosphate, increased over time. In contrast, the peritoneal clearances of TMAO, IS, PCS, and β2-microglobulin (β2M) did not change significantly, while IL-6 showed a downward trend (Ji et al., 2025). In addition, longitudinal studies suggest that the loss of residual kidney function is associated with the accumulation of IS and PCS, whereas switching from CAPD to high-volume automated peritoneal dialysis (APD) does not clearly enhance their removal (Eloot et al., 2015). Another longitudinal study showed that the higher total solute clearance observed in patients receiving incremental peritoneal dialysis (IPD) may be more related to better preservation of residual kidney function, rather than to enhanced peritoneal clearance of IS, PCS, β2M, or IL-6 (Ji et al., 2025). Therefore, in PD patients, preservation of residual kidney function, maintenance of peritoneal membrane function, reduction of gut-derived toxin generation, and, in selected cases, transition to or combination with extracorporeal blood purification strategies may be more relevant for controlling PBUT burden than simply intensifying the PD prescription (Figure 2).

## 5 Conclusion and outlook

Dialysis is an essential life-sustaining treatment for patients with kidney failure, and its clinical importance cannot be replaced. Cognitive impairment is common in dialysis patients, but its underlying mechanisms remain insufficiently understood. Current evidence suggests that dialysis itself is an important driving factor for cognitive impairment. The microbiota–gut–kidney–brain axis provides a new integrative framework for understanding cognitive impairment in dialysis patients. Dialysis patients often show gut dysbiosis, accumulation of gut-derived uremic toxins, reduction of protective metabolites such as SCFAs, increased LPS burden, bile acid dysregulation, and impaired intestinal barrier function. These changes may be linked to systemic inflammation, oxidative stress, endothelial dysfunction, BBB injury, and neuroinflammation. Therefore, gut microecology and its metabolites may serve as important intermediate links connecting the uremic state, dialysis-related stress, and neurocognitive injury.

Based on current evidence, gut microecological changes in dialysis patients show a relatively consistent direction. These changes include decreased beneficial commensal bacteria, increased opportunistic pathogens or potential toxin-producing bacteria, reduced *α*-diversity, and accumulation of gut-derived UTs, such as IS, PCS, IAA, Hcy, TMAO, and PAG. They are also accompanied by bile acid dysregulation, decreased protective metabolites such as SCFAs, increased inflammatory factors, including IL-6, TNF-α, IL-1β, and CRP, and increased LPS burden. However, the strength of evidence differs among different UTs. For example, the relationship between TMAO and cognitive impairment may be confounded by kidney clearance, diet, cardiovascular risk, and inflammatory status. Although IS has relatively more experimental evidence supporting its neurotoxicity, its independent role in cognitive decline among dialysis patients still requires further validation. The relative contributions of PCS, PAG, hippuric acid, and other metabolites also remain unclear. Moreover, direct evidence regarding the relationship between gut microbiota, microbial metabolites, and cognitive impairment in dialysis patients remains limited. Most clinical studies are cross-sectional or observational and cannot establish causality. Many findings are mainly derived from animal models or *in vitro* experiments and cannot be directly extrapolated to the complex dialysis population. In addition, studies on the specific effects of HD and PD on gut microbiota remain limited. HD-related changes may mainly be associated with intermittent extracorporeal circulation, intradialytic hemodynamic fluctuations, intestinal hypoperfusion, inflammatory responses, and periodic changes in uremic toxin levels. In contrast, PD-related changes may be more closely related to long-term glucose dialysate exposure, chronic peritoneal inflammation, peritoneal fibrosis, the risk of peritonitis, and ultrafiltration failure. These dialysis modality-related factors may alter the gut microenvironment and metabolic profiles, but their independent effects on cognitive outcomes still require further investigation. Animal studies of stroke have shown that gut-derived bacteria may translocate through the circulation and reach organs such as the brain (Peh et al., 2026). This suggests that, in patients with dialysis-associated cognitive impairment, translocation of gut-derived bacteria or microbial components into brain tissue may also represent a potential pathological mechanism.

With the development of multi-omics technologies, spatial transcriptomics, spatial proteomics, spatial metabolomics, single-cell transcriptomics, single-cell proteomics, single-cell metabolomics, and third-generation long-read strain-level metagenomics may help clarify the potential mechanisms of cognitive impairment in dialysis patients from the perspectives of tissue spatial organization, cellular heterogeneity, and strain-level functional differences. Meanwhile, gut microecological research should not be limited to bacteria. It should also be extended to archaea, fungi, bacteriophages, viruses, protozoa, and other microbial components, with greater attention to their multi-kingdom interactions. In addition to bacteria, these microbial components may also participate in gut–brain axis-related processes by regulating bacterial community structure, metabolic function, intestinal barrier status, and host immune responses. Existing studies suggest that gut *methanogenic archaea* are associated with cognitive performance (Fumagalli et al., 2025); gut virome and bacteriophage signatures may be linked to early Alzheimer’s disease (AD) pathology or cognitive impairment (Ghorbani et al., 2023); and the gut mycobiome is also considered an emerging factor worthy of attention in AD and related cognitive disorders (Nagpal et al., 2020). In addition, gut single-cell eukaryotes such as *Blastocystis* have been associated with impaired executive function (Mayneris-Perxachs et al., 2022). However, current evidence mainly comes from healthy populations, Mild cognitive impairment (MCI)/AD cohorts, or animal studies, and direct validation in dialysis patients is still lacking. Therefore, future studies should combine long-read metagenomics, viromics, mycobiomics, and protistomics to systematically evaluate interactions among different microbial kingdoms and their relationship with cognitive impairment in dialysis patients.

Previous studies have shown that dialysis cannot sufficiently reduce all types of PBUT (Saar-Kovrov et al., 2021). Therefore, gut microecological regulation is considered a direction with translational potential. Plant-based dietary patterns, intradialytic exercise training, supplementation with probiotics and related preparations, FMT, targeted reduction or clearance of gut-derived uremic toxins and related metabolites, and extracorporeal blood purification strategies combined with HD or PD may restore microbial homeostasis, improve gut barrier function, and reduce gut-derived UT burden, thereby producing potential beneficial effects on cognitive function. However, current evidence is mainly derived from animal models, and clinical studies usually have small sample sizes. Future studies should conduct high-quality randomized controlled trials with larger sample sizes, clear stratification strategies, and cognition- and function-related outcomes as primary endpoints.
